# Quality over quantity: joint group dance strengthens schoolchildren's social connections and wellbeing

**DOI:** 10.3389/fcogn.2026.1783852

**Published:** 2026-06-04

**Authors:** Bahar Tunçgenç, Chloe Wider, Katie Spreadborough, Kaleab A. Kinfu

**Affiliations:** 1 Psychology Department, Nottingham Trent University, Nottingham, United Kingdom; 2 Freedom Foundation UK Ltd., Nottingham, United Kingdom; 3 Center for Innovation in Data Engineering and Science, University of Pennsylvania, Philadelphia, PA, United States

**Keywords:** children, creative health, mental wellbeing, school-based intervention, social connection

## Abstract

**Background:**

Children are experiencing high levels of loneliness and mental health problems around the globe, with those from underserved communities being affected disproportionately. Group dance programmes, which facilitate social connections and wellbeing, can buffer against loneliness. However, we know little about the mechanisms behind the effectiveness of these programmes and their long-term and group-level effects. This study examined whether it is mere engagement in group dance or better individual dance performance that fosters social connections and wellbeing.

**Methods:**

A pre-post design was used to assess the social connection and wellbeing effects of a 10-week-long group dance programme in schoolchildren (*N* = 25) from a low-income background. Social connection was measured at peer-to-peer and whole-class network levels. Dance performance was measured using state-of-the-art motion tracking techniques. Wellbeing was measured using the well-established KIDSCREEN-10 questionnaire.

**Results:**

Linear mixed effects models and Social Network Analysis were conducted to examine change over time. Participation in group dance, but not dance performance, was associated with significant increases in reciprocal friendships and reciprocal best friends. Wellbeing improved over time, and an increase in friendship quality (i.e., reciprocal friendships), but not just quantity (i.e., social network size), predicted improvements in wellbeing.

**Conclusions:**

Joint group dance in a school setting can enhance wellbeing at individual-level and strengthen friendship quality at peer-level and whole-class level. These positive changes are observed regardless of individuals' performance, indicating that the joint nature of group dance can be sufficient to elicit social and wellbeing benefits.

## 1 Introduction

Mental health difficulties affect 17.4% of young people in the UK, many of whom show symptoms by age 16 (NHS Digital, 2021). This is strongly linked to loneliness, with children with probable mental illnesses being twice as likely to feel lonely most or all of the time (NHS Digital, 2021). Globally, there is increasing acknowledgment that the arts can play a crucial, preventative role in promoting mental and physical health through enhancing social connections and reducing loneliness (Dow et al., 2023). In this study, we examined how a school-based arts programme involving group dance can enhance children's social connections with their peers and mental wellbeing.

Supportive friendships are also widely recognized as protective factors against loneliness and mental health risks within school settings, where peer relationships are pivotal (Pollak et al., 2023). School-based interventions designed to promote friendships and social-emotional learning have become increasingly common, with many interventions focusing on individual skill-building rather than seeking to change peer network dynamics (Manchanda et al., 2023; Pollak et al., 2024). Recent reviews note that these individually focused approaches may not have a substantial or lasting impact on classroom climate or friendship networks (Cahill and Dadvand, 2020; Pollak et al., 2023). Instead, whole-class interventions that target social participation, inclusion, and mental wellbeing can be beneficial at a larger scale, especially with non-clinical populations (Cappella et al., 2013; Durlak et al., 2011). For example, whole-class interventions have been shown to increase inclusive group play and strengthen children's social-emotional competencies, according to teacher reports (Hassani et al., 2023). Similarly, interventions promoting classroom norms and collaborative activities can foster more supportive and inclusive environments (Patalay et al., 2020; Sterrett et al., 2017).

Engagement in arts-based interventions can enhance social connections and sustain mental health through various psychological, biological, social, cognitive and behavioral mechanisms (Fancourt and Finn, 2019). Engaging in group dance can enhance participants' sense of joint action and cooperation, enjoyment, group performance (Kirschner and Tomasello, 2009) and bonding, subsequently contributing to better wellbeing and a positive future outlook (Tunçgenç et al., 2024). Myriad lab-based studies show that social connection and pro-social behaviors increase after participants imitate (i.e., do the same moves) and synchronize (i.e., at the same time) with each other (Mogan et al., 2017; Over and Carpenter, 2013; Rennung and Göritz, 2016). In group dance, performing the same movements at the same time can trigger mirroring mechanisms (Crone et al., 2021; Wang and Hamilton, 2012), resulting in enhanced closeness and connection among participants. Engaging in joint activities can signal group membership and trigger social norm-based decision-making processes, especially when the activities involve imitation and synchrony (Legare and Nielsen, 2015). For instance, 8- to 11-year-olds who did the same dance moves in synchrony with members of another group showed greater social connection and reduced out-group biases compared to those who did a different dance out of sync (Tunçgenç and Cohen, 2016). This line of work has revealed the specific role of imitation and synchrony in enhancing social connections. However, these variables rarely manifest as clean-cut as in laboratory study designs. Thus, further work is needed to understand whether mere participation in these activities could be sufficient to elicit the observed benefits in real-world settings.

Outside laboratory settings, arts-based interventions incorporating imitation/synchrony as a core feature have been shown to improve wellbeing, particularly by enhancing social connections. Group singing has been found to increase self-reported closeness and positive affect more effectively than non-synchronous creative activities such as crafts or writing (Pearce et al., 2015). A systematic review reported that in naturalistic settings, group singing promoted wellbeing through increased social connection and self-confidence (Glew et al., 2020). Interventions involving group music and dance can be promising for children from disadvantaged backgrounds, who experience disproportionately high levels of loneliness and mental health difficulties (Kumar et al., 2018; Siva, 2020). A 10-week, school-based group drumming intervention for disadvantaged adolescents in Australia called DRUMBEAT led to significant improvements in mental wellbeing, reduced post-traumatic stress symptoms, and decreased antisocial behavior among boys (Martin and Wood, 2017). Another study found that 11- to 12-year-old DRUMBEAT participants who were shy or socially withdrawn had particularly increased self-esteem, school attendance and cooperation post-intervention (Faulkner et al., 2012). In the area of dance interventions, Rajan and Aker (2020) showed that a weekly, year-long group dance intervention for at-risk 3–5-year-olds in California improved social-emotional competence, including peer relationships and self-identity.

Despite providing valuable insights, these intervention studies leave unanswered whether the observed benefits arise from imitation/synchronization accuracy or merely from the joint group activity. Participating in a collective activity alone can foster connection, regardless of how closely the participants align with each other in space and time (Wilson et al., 2018). Therefore, both imitation/synchronization accuracy and shared group participation may serve as complementary pathways to social connection and wellbeing (Basso et al., 2021; Bieńkiewicz et al., 2021). It is important to understand these mechanisms for effective, scalable and consistent roll-out of such interventions. We also still know little about the longitudinal effects of school-based music and dance interventions, especially in socially and economically disadvantaged communities, who often need support the most (van der Graaf et al., 2024). To explore these dynamics, this study combined children's longitudinal self-report survey responses on wellbeing and social networks with behavioral motion-tracking data to examine whether (1) a group dance programme promoted children's social connections and mental wellbeing, (2) children who had closer social connections had better mental wellbeing, and (3) children with better dance performance (i.e., through imitating/synchronizing with their teacher better) had closer social connections.

## 2 Methods

### 2.1 Participants

Twenty-five Year-3 pupils aged 7–8 years old (9 boys, 16 girls) were recruited from the Henry Whipple Primary School in Nottingham, UK from March—May 2023. The participants were from the same class and were enrolled in the Freedom Foundation UK's school-based programme Freedom Factory, designed to support children's emotional wellbeing, self-worth, and coping skills through music and dance. Participation in the study was independent of participation in the dance programme. The school was located in Bestwood, one of the top 20% most deprived areas within England (Ministry of Housing Communities and Local Government, 2019), with 49.7% of the pupils in the school receiving free school meals (Department for Education, 2025), a figure well above the national average of 24.3% for state-funded primary schools in England at time of data collection (Department for Education, 2024). According to parental report, the participants' ethnicities were: 3 Asian/Asian British, 6 Black, African, Caribbean or Black British, 5 mixed or multiple ethnic groups, 5 White, and 6 preferred not to say. One additional child, whose parents consented to the study, had an autism diagnosis, and often missed part or all of the sessions and was therefore excluded from analyses.

Written parental consent and verbal assent from children were obtained before data collection. One child, whose parent declined video consent, was excluded from dance performance analysis. The study was reviewed by Nottingham Trent University's Business, Law and Social Sciences Research Ethics Committee.

### 2.2 Design

**Figure 1:**
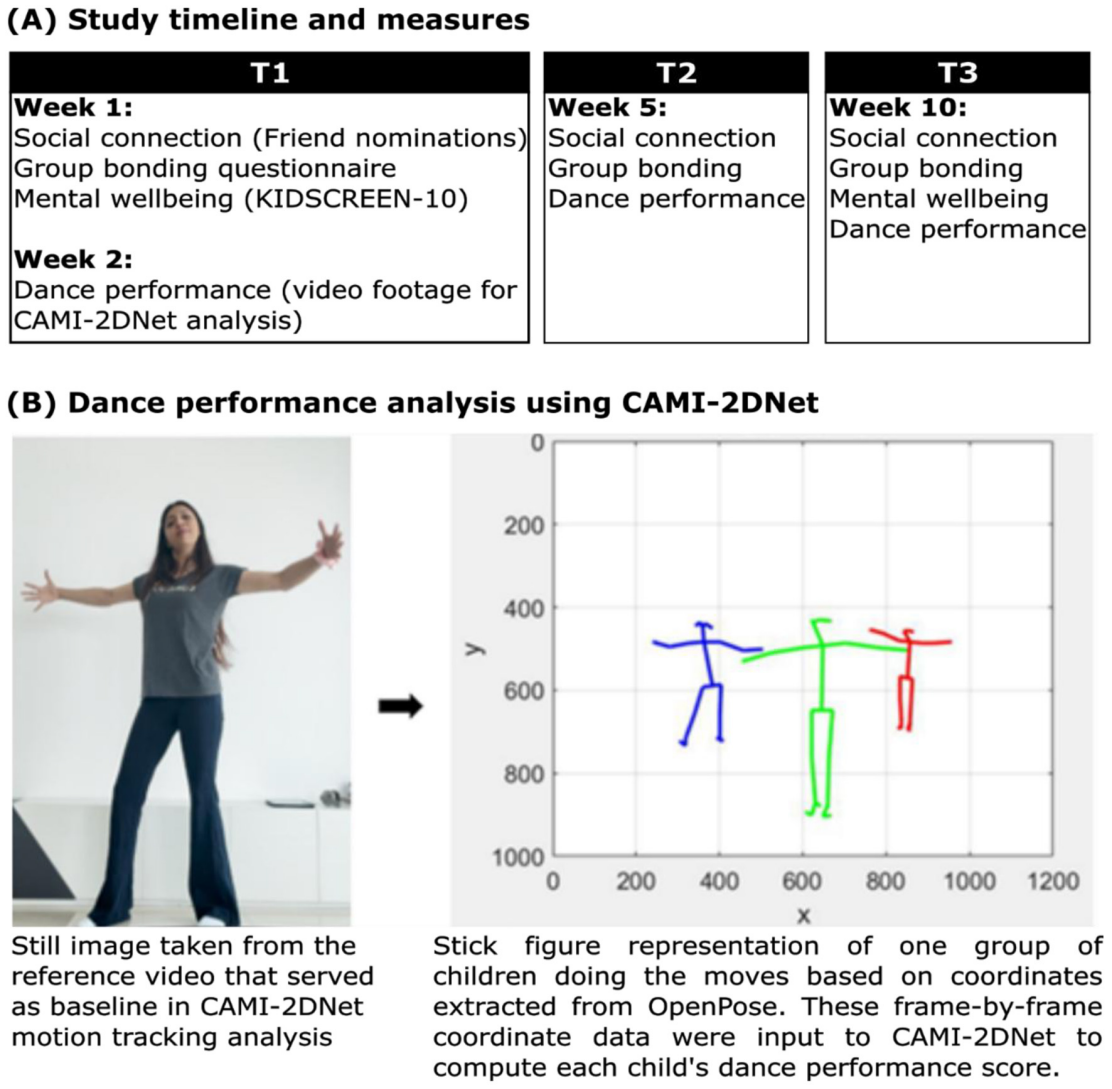
Overview of study methods. **(A)** Study timeline and measures collected at each timepoint (T1–T3), and **(B)** schematic summarising CAMI-2DNet motion tracking analysis to assess children's dance performance.

The study employed a longitudinal design spanning a 10-week whole-class music and dance programme. Measures for social connection, group bonding, and dance performance were collected at three time-points: before the programme started (T1), at midpoint (T2), and after the programme ended (T3; Figure 1). Wellbeing was measured at T1 and T3. Social connection and wellbeing surveys were administered in the children's classroom, with accommodations provided for children requiring additional reading support. Dance performance was measured post-hoc via video-recordings obtained during the sessions.

### 2.3 Measures and procedure

#### 2.3.1 The programme

Two professional dancers from the Freedom Foundation UK facilitated 10 weekly sessions that lasted 1.5 h each. A researcher was also present to note any events that could impact programme delivery and/or the children's performance (none was recorded). Systematic assessment of delivery fidelity was not conducted due to resource constraints. All 25 children attended all sessions. Each session opened with a warm-up group activity (e.g., Simon Says), followed by a circle time discussion, during which a specific theme related to mental health and wellbeing was explored (i.e., acceptance, self-worth, managing anxiety, online safety on social media, and kindness). Next, the children practiced a dance choreography, which they performed in the final week of the programme to their parents and teacher. Every other week, the children also engaged in a song writing and singing activity, during which they wrote positive affirmations related to that week's theme (e.g., “I am confident”, “I am resilient” etc.).

#### 2.3.2 Social connection

Children's friend nominations and a group bonding questionnaire assessed social connection. For friend nominations, children first selected which of the other participants they considered a friend and then marked which top three were their best friends. These data were used to derive peer-to-peer social connection metrics of how many friends each child nominated, how many times they were befriended by others, how many reciprocal friends and reciprocal best friends they had, and degree centrality, indicating how popular a child was. Social connection within the whole-class network was assessed using SIENA (Simulation Investigation for Empirical Network Analysis) modelling metrics of outdegree, reciprocity, and transitive triplets (details below).

In addition, social connection within small groups was assessed using the group bonding questionnaire. To this end, the children were randomly assigned to eight groups of three by the researchers on Week-1. These groups danced in close proximity during the sessions and were video-recorded together to assess dance performance (Figure 1). In the group bonding questionnaire, children rated how much their group members: (i) helped each other with a difficult task, (ii) told secrets to each other, and (iii) lent things that they needed to each other (1 = Never to 5 = Always). Children also completed the Identity Fusion Scale (Swann et al., 2009), a single-item pictorial measure, in which two circles representing the participants and their group were shown in gradually increasing proximity to each other (1 = distant, non-overlapping circles to 5 = self completely subsumed within group) and asked to select the image that best represents how close or distant they felt to their group. The scores from these four items were averaged to produce an overall group bonding score, with higher scores indicating stronger connection.

#### 2.3.3 Mental wellbeing

Self-report KIDSCREEN-10 questionnaire, normed across European Union countries, including the U.K., for measuring child and adolescent wellbeing, assessed wellbeing (Ravens-Sieberer et al., 2010). Children responded using a 5-point Likert scale (1 = “never” or “not at all”, 5 = “always” or “very much”), reflecting on their experiences over the past week. Following scale instructions, item responses were summed to produce a raw score ranging from 10 to 50, with higher scores indicating better quality of life. Ravens-Sieberer et al. (2010) reported good internal consistency (Cronbach's alpha= 0.82) and test-retest reliability (*r* = 0.73; ICC = 0.72). The average T-score for our sample, based on UK KIDSCREEN-10 norms for 8–11 years was 45.10, indicating lower wellbeing in our sample than the mean of the reference population (T = 50; Ravens-Sieberer et al., 2010).

#### 2.3.4 Dance performance

Dance performance was quantified using CAMI-2DNet as the motion similarity between a reference video (i.e., of a dancer doing the moves) and each child's video recorded at 30 fps (Kinfu et al., 2025). To this end, post-hoc motion tracking was conducted on video recordings taken as the children danced within their randomly assigned groups of three (or two, in cases of absence). The first video footage was taken on Week-2 to allow children time to familiarize themselves with the moves. For the recordings, the groups danced in a designated area away from the class to minimize obstructions. One of the moves in the choreography involved participants crossing paths, which led to identity confusion during motion tracking. Therefore, the first 10 s of the choreography preceding this crossover were retained for analysis, which included three moves comprising upper body movements.

For analysis, we first extracted frame-by-frame 2D coordinates of five keypoints (torso, left arm, right arm, left leg, and right leg) using OpenPose (Ino et al., 2024). Since OpenPose does not preserve person identity between frames, each individual's keypoints were matched across consecutive frames using the Hungarian algorithm (linear sum assignment), minimizing the Euclidean distance between corresponding pose vectors (Gan et al., 2021; Lee et al., 2022). Where OpenPose detected bodies not corresponding to any participant, these were removed. We input the 2D coordinates to the CAMI-2DNet encoder network, which has been trained to capture intrinsic characteristics of body movements by isolating motion dynamics from nuisance factors such as variations in body shape and camera viewpoint. CAMI-2DNet calculated cosine similarity between the reference and child's videos per keypoint, which we aggregated using a weighted average to compute a single dance performance score for each child. The weight of a keypoint was the average, normalized displacement of that keypoint for that dance move in the teacher's reference video. This approach ensured that body parts with significant movements had a proportionally greater influence on the final dance performance score.

### 2.4 Data analysis

To examine Research Question 1, linear mixed-effects models with participant ID as a random intercept were used, testing for within-subject changes across timepoints in social connection, dance performance, and wellbeing. The random effect variance in the wellbeing model was zero, indicating no detectable individual differences after accounting for timepoint and resulting in a reduced standard linear model. Similarly, linear mixed effects models examining Research Questions 2 and 3 revealed zero variance; thus, regression models were used assessing the associations between change scores. The models exploring the associations between dance performance and social connection for Research Question 3 included a fixed effect for dance performance that was grand-mean centered due to the narrow range of children's dance performance scores.

To examine longitudinal changes in network-level social connection, a Stochastic Actor-Oriented Model (SAOM) was estimated using the SIENA package in R (Ripley et al., 2026; Snijders, 2017). Unlike traditional regression methods, SIENA does not report *p*-values. Instead, statistical significance is inferred when the ratio of the estimate to its standard error exceeds 2.0, following conventions based on standard normal approximations (Snijders et al., 2010) nominations data collected across three timepoints were formatted as a 3D adjacency matrix, where each cell represented the presence (1) or absence (0) of a directed friend nomination. The model included two time periods (period 1: T1–T2, period 2: T2–T3) and focused on three effects: outdegree (also referred to as density), capturing the baseline tendency to form friendship ties, reciprocity, the tendency for actors to return friendship nominations, and transitive triplets, triadic closure or the likelihood that a friend of a friend becomes a friend.

## 3 Results

### 3.1 Change over the course of the programme

**Figure 2:**
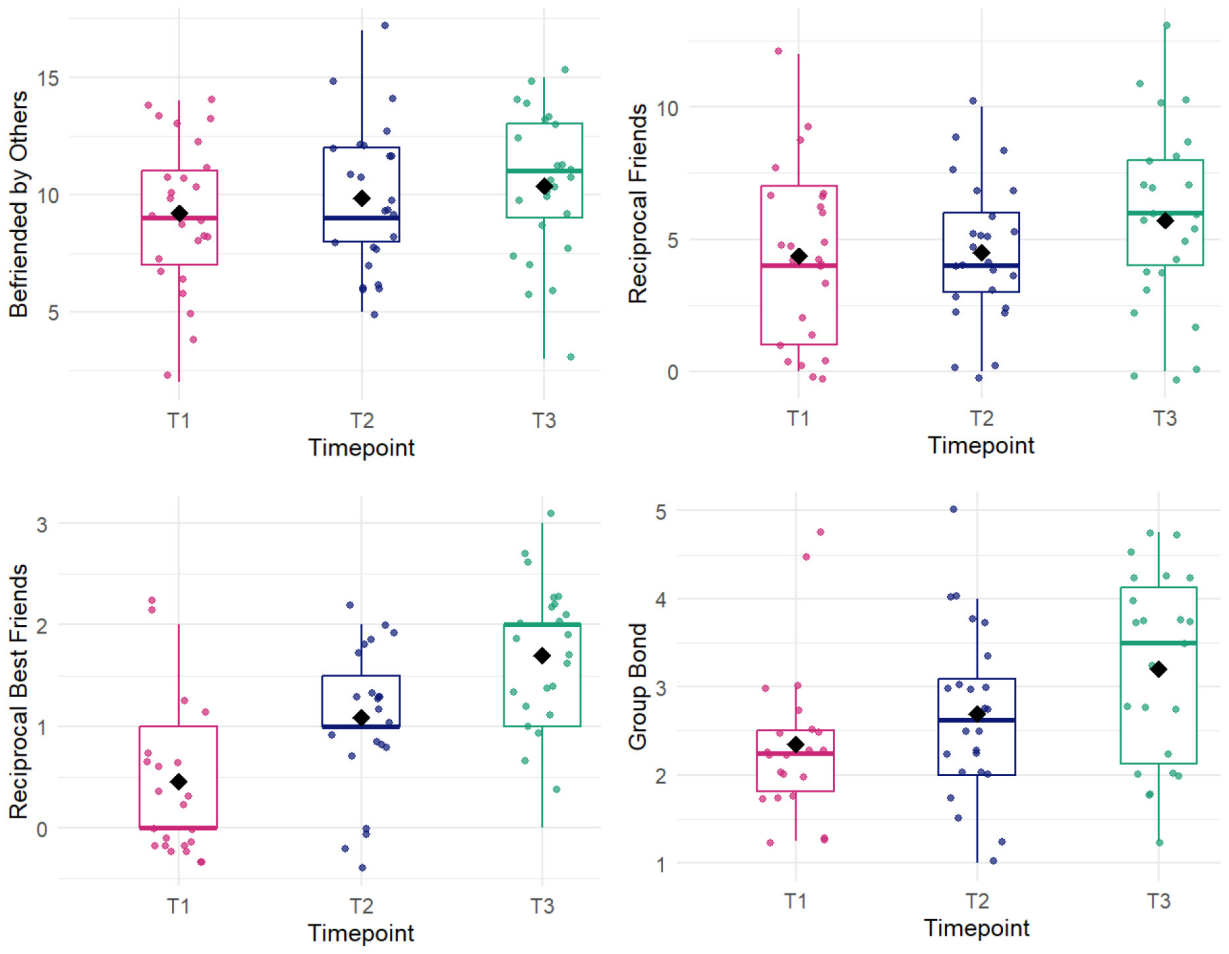
Boxplots displaying significant changes in children's self-reported social connection across timepoints (T1–T3). In all box plots, the colorful dots indicate individual data points, the black diamonds indicate group mean, the horizontal lines indicate group median, and whiskers indicate the interquartile range.

A significant increase was found from T1 to T3 in how many times the participants were befriended by others (*b* = 1.16, *p* = 0.02), number of reciprocal friends (*b* = 1.36, *p* = 0.006), number of reciprocal best friends (b = 1.24, *p* > 0.001) and group bonding (b = 0.85, *p* = 0.005; Figure 2). Only reciprocal best friends significantly increased from T1 to T2 (b = 0.62, *p* = 0.001). No difference across timepoints was observed for the number of friends (b = 1.16, *p* = 0.23), and degree centrality (b = 0.11, *p* = 0.14; see Supplementary Tables S1–S3 for full model outputs).

**Figure 3:**
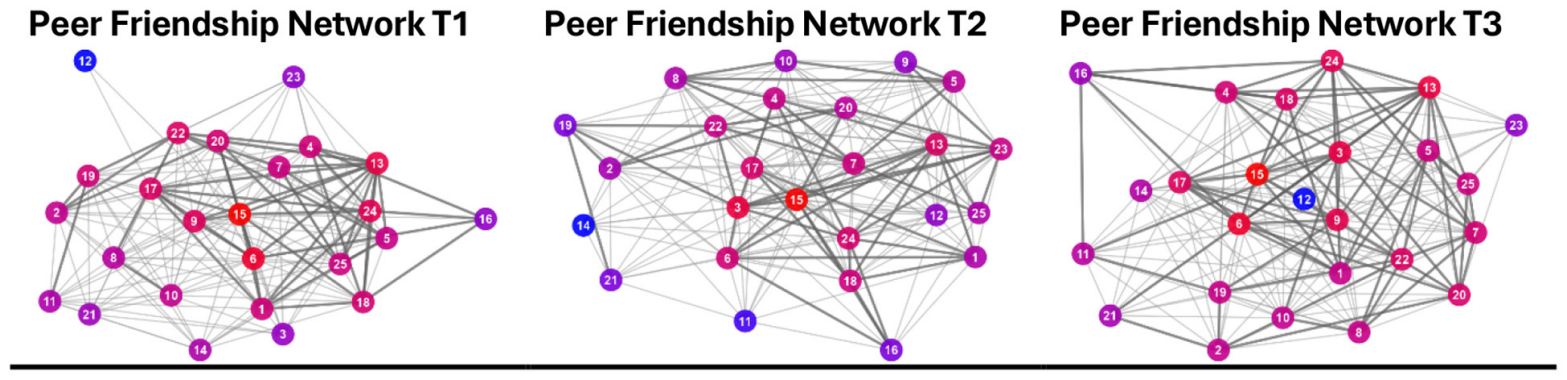
Undirected network graphs illustrating friend nominations among participants at each timepoint (T1–T3). Each node represents one participant (*N* = 25). Lines between nodes indicate a friend nomination between those participants. Thicker lines represent reciprocal friendships, while thinner lines indicate unidirectional nominations. Node color intensity corresponds to degree centrality, calculated as the total number of incoming and outgoing friend nominations (blue = fewer nominations, red = more nominations). Node position reflects force-directed layout for visual clarity and does not imply structural centrality. Node color represents total degree (number of outgoing and incoming friend nominations made).

Regarding the social network analysis (Figure 3), the SAOM model converged adequately after 1,871 iterations, with a maximum convergence ratio of 0.10, below the standard threshold of 0.25, indicating acceptable stability in parameter estimates. The rate parameters reflect the estimated number of opportunities actors had to make changes to their friendship ties between timepoints. The rate was higher in period-1 (estimate= 11.04, SE= 1.37) than in period-2 (estimate= 8.25, SE= 0.88), indicating that the network stabilized over time. The outdegree effect was significantly negative (estimate = −1.01, SE = 0.10), indicating overall network sparsity, i.e., participants tended to form relatively few friendship ties. Reciprocity was small and non-significant in period-1 (estimate= 0.09) but increased in period-2 (interaction estimate = 0.44), approaching significance, suggesting a possible strengthening of reciprocal friendships over time, though the effect did not reach conventional significance. Transitive triplets were strong in period-1 (estimate = 0.11) and showed a non-significant decline in period-2 (interaction estimate = −0.02), indicating that triadic closures persisted across time.

Regarding change in dance performance, there was a significant decrease from T1 to T2 [b = −0.017, *p* = 0.04, 95% CI (−0.033, −0.001) and a non-significant increase at T3 (b = −0.014, *p* = 0.11], indicating that scores dipped mid-way and partially returned to baseline by T3. Lastly, there was a statistically significant increase in wellbeing from T1 to T3 (b = 0.27, SE= 0.10, t_(42)_= 2.88, *p* = 0.006).

### 3.2 Associations between change in social connection and mental wellbeing

Having more reciprocal friends was significantly associated with higher wellbeing (b = 0.12, *p* = 0.006), with this association being weaker at T3 than T1 (b = −0.18, *p* = 0.004). No other social connection metric significantly predicted wellbeing (number of friends: b = 0.03, *p* = 0.22, befriended by others: b = 0.05, *p* = 0.31; reciprocal best friends: b = 0.25, *p* = 0.163; degree centrality: b = 0.30, *p* = 0.39), and neither did they significantly interact with timepoint.

### 3.3 Associations between change in social connection and dance performance

There was a significant effect of dance performance at T1 on reciprocal best friends (b = 15.27, *p* = 0.035), such that better dance performance was associated with a higher number of reciprocal best friends. There was also a significant effect of dance performance on number of friends at T1 (b = −65.52, *p* =0.047), with poorer dance performance being associated with a higher number of friends. There was no significant effect of dance performance on the other social connection metrics (befriended by others: b = −5.07, *p* =0.74; reciprocal friends: b = −27.73, *p* = 0.069; degree centrality: b = −2.79, *p* = 0.22). Significant effects of timepoint were also observed, consistent with the increases in social connection reported in Part 1. There was no significant interaction effect of dance performance and timepoint on any of the social connection metrics (all *p* >0.10). These results indicate that change in dance performance from T1 to T3 did not reliably predict changes in social connection outcomes.

## 4 Discussion

Using a pre-post design, we examined the social connection and wellbeing benefits of a 10-week-long music and dance intervention in schoolchildren. Social connection was measured at peer-to-peer, group, and whole-class network levels. Participation in the intervention increased reciprocal friendships and reciprocal best friendships, suggesting the programme fostered meaningful, mutually recognized relationships. This improvement in social connection was paralleled by an increase in children's wellbeing over the intervention period. Notably, reciprocal friendships predicted improvements in wellbeing. We did not find an association between the children's dance performance and social connection.

The fact that reciprocal friendships, but not mere number of friends or popularity, were linked to improvements in mental wellbeing speaks to the importance of positive quality friendships beyond simply having a larger friend network. Whilst previous research has similarly suggested that more integrated peer networks provide stronger social support than exclusive dyads in adults (McNamara et al., 2021) and older children (Turetsky et al., 2020), how friendship quality contributes to wellbeing in young children is less well-understood. This is partly due to inherent challenges in measuring changes in wellbeing, which is a complex, multi-factorial construct (Ryff, 1989). In theory, a child can have high quality friendships, and still wellbeing may not be impacted if, for instance, they are struggling with other aspects of wellbeing such as purpose in life or environmental mastery, which improvements in positive relationships may not directly impact upon. The nature and characteristics of a child's peer group matter also. For instance, close friendships with peers who exhibit antisocial behavior or poor emotional regulation may not support, and may even undermine, a child's wellbeing (Dishion and Tipsord, 2011). In this study, we assessed mental wellbeing holistically, considering these multi-factorial dimensions. Future research can tease apart the relative contributions of each dimension and/or the children's peer group dynamics to better understand how social interactions can support mental wellbeing.

While direct evidence linking classroom cohesion to wellbeing in primary school is limited, related constructs such as peer support and school belonging have consistently been shown to predict mental wellbeing in children (Butler et al., 2022; Parr et al., 2020). Similarly, interventions that target collective participation have been shown to foster more supportive and inclusive school climates (Hassani et al., 2023; Patalay et al., 2020). In our study, increased reciprocal friendships over time might represent greater integration and a more supportive classroom network. Our findings on increased group bonding and network density further indicate that improvements in social connection extended beyond pairs, enhancing whole-class cohesion. This goes beyond what is typically captured by individual-focused interventions, supporting the argument that whole-class approaches can reshape the broader social environment.

Importantly, these social and wellbeing effects were independent from children's dance performance, which showed a mid-intervention decline with subsequent recovery. It is possible that our measure of dance performance, which relied on accurate imitation, was too conservative to capture the kind of social synchrony that fosters friendships. Additionally, simply engaging in a shared, novel activity can foster bonding among children, especially in primary school. Research shows that (Haj-Mohamadi et al., 2018; Tunçgenç and Cohen, 2016) participating in a structured activity like music and dance provides a predictable framework for repeated, shared interactions, which are key ingredients in friendship formation. Beyond exact synchrony, group music and dance have been shown to promote a sense of unity, shared purpose, and affiliation (Lee et al., 2020; Pearce et al., 2015). It is therefore possible that the affective and collective nature of our programme played a more central role in increasing classroom social ties than individual performance.

Because the intervention was embedded within the school day, all children participated regardless of prior interest, ability, or social status. This contrasts with after-school clubs, which often attract already socially confident or high-performing children, amplifying existing inequalities (Mahoney et al., 2005). The classroom-wide format of this programme likely increased exposure between children who might not otherwise interact, thus supporting new or broader peer connections. Thus, we would recommend future interventions aiming to enhance classroom cohesion and elicit new, mutual friendships to be integrated into the curriculum rather than offered as optional add-ons.

This study has four main limitations. First, participants were drawn from a single school and age group within a specific socio-economic context, so results may not extend to other cultural or educational settings. This also meant that there was no control or comparison group, limiting the causal links we can draw. Second, we used self-report and fixed-choice sociometric nominations (e.g., forced choice of maximum three “best friends”). Such designs can restrict the upper bound of ties, are sensitive to short-term social dynamics, and may under-detect more diffuse changes in peer relations (Neal, 2024; Poulin and Dishion, 2008). Third, unmeasured classroom and school-level characteristics (e.g., teacher warmth, peer conflict, broader school climate) could influence outcomes independently of the program; variation in adult facilitation was not controlled and may have shaped programme effectiveness (Roubinov et al., 2020; Wang and Degol, 2016). Additionally, variation in pedagogy, which can shape delivery and potentially introduce bias, was not controlled (Durlak and DuPre, 2008). Finally, the absence of a control group, the three-time-point structure, and lack of a longer-term follow-up limit our ability to make causal inferences, model trajectories, and assess the persistence of any effects observed.

This study provides evidence that a classroom-based music and dance programme can enhance both social connection and wellbeing in primary school children. Wellbeing increased over the course of the program, alongside multiple indicators of social connection; notably, only reciprocal friendships were linked to increased wellbeing, underscoring the importance of mutual, high-quality ties. Increased classroom network density further suggests that the intervention fostered social integration beyond dyadic friendships. These findings indicate that whole-class creative activities may be a promising approach to supporting social-emotional development and wellbeing in children.

## Data Availability

The datasets presented in this study can be found in online repositories. The names of the repository/repositories and accession number(s) can be found below: https://osf.io/pru9n/.
