# Supplementary material for: Quality over quantity: joint group dance strengthens schoolchildren's social connections and wellbeing
Source: Front Cognit. 2026 Jun 4;5:1783852. doi: 10.3389/fcogn.2026.1783852 (PMC13271117; doi:10.3389/fcogn.2026.1783852)
Supplement: Supplementary file 1 [file Table_1.docx]

**Quality over Quantity: Joint Group Dance Strengthens Schoolchildren’s Social Connections and Wellbeing**

**Supplementary Information**

| *Table S1*  *Outputs of linear mixed-effects models predicting social connection outcomes (number of friends, befriended by others, reciprocal friends, reciprocal best friends, degree centrality, and group bond) across timepoints (T1 – T3).* | | | | |
| --- | --- | --- | --- | --- |
| **Outcome** | **Predictor** | **Estimate** | **Std. Error** | **p-value** |
| **Friends** | Intercept (T1) | 9.20 | 1.28 | > 0.001 |
|  | T2 vs T1 | 0.64 | 0.97 | 0.511 |
|  | T3 vs T1 | 1.16 | 0.97 | 0.236 |
| **Befriended by others** | Intercept (T1) | 9.20 | 0.62 | > 0.001 |
|  | T2 vs T1 | 0.64 | 0.50 | 0.202 |
|  | T3 vs T1 | 1.16 | 0.50 | 0.023* |
| **Reciprocal friends** | Intercept (T1) | 4.36 | 0.64 | > 0.001 |
|  | T2 vs T1 | 0.12 | 0.47 | 0.799 |
|  | T3 vs T1 | 1.36 | 0.47 | 0.006** |
| **Reciprocal best friends** | Intercept (T1) | 0.46 | 0.15 | 0.003 |
|  | T2 vs T1 | 0.62 | 0.18 | 0.001** |
|  | T3 vs T1 | 1.24 | 0.18 | > 0.001*** |
| **Degree centrality** | Intercept (T1) | 0.98 | 0.08 | > 0.001 |
|  | T2 vs T1 | 0.03 | 0.07 | 0.722 |
|  | T3 vs T1 | 0.11 | 0.07 | 0.147 |
| **Group bonding** | Intercept (T1) | 2.36 | 0.21 | > 0.001 |
|  | T2 vs T1 | 0.33 | 0.28 | 0.246 |
|  | T3 vs T1 | 0.85 | 0.29 | 0.005** |

| *Table S2*  *Outputs of linear mixed-effects models predicting mental wellbeing as a function of social connection metrics (number of friends, befriended by others, reciprocal friends, reciprocal best friends, and degree centrality) at T1 and T3, including coefficients for their main effects and interactions with timepoint.* | | | | |
| --- | --- | --- | --- | --- |
| **Outcome** | **Predictor** | **Estimate** | **Std. Error** | **p-value** |
| **Mental wellbeing** | Intercept | 3.15 | 0.27 | > 0.001 |
|  | Friends | 0.03 | 0.02 | 0.218 |
|  | Timepoint (T3) | 1.00 | 0.40 | 0.016* |
|  | Friends x Timepoint (T3) | -0.04 | 0.03 | 0.197 |
| **Mental wellbeing** | Intercept | 2.99 | 0.46 | > 0.001 |
|  | Befriended by others | 0.05 | 0.05 | 0.306 |
|  | Timepoint (T3) | 0.59 | 0.72 | 0.417 |
|  | Befriended x Timepoint (T3) | -0.01 | 0.07 | 0.896 |
| **Mental wellbeing** | Intercept | 2.86 | 0.23 | > 0.001 |
|  | Reciprocal friends | 0.12 | 0.04 | 0.006** |
|  | Timepoint (T3) | 1.49 | 0.36 | >0.001*** |
|  | Reciprocal friends x Timepoint (T3) | -0.18 | 0.06 | 0.004** |
| **Mental wellbeing** | Intercept | 3.33 | 0.14 | > 0.001 |
|  | Reciprocal best friends (T1) | 0.25 | 0.18 | 0.163 |
|  | Timepoint (T3) | -0.30 | 0.32 | 0.349 |
|  | Reciprocal best friends x Timepoint (T3) | 0.32 | 0.23 | 0.180 |
| **Mental wellbeing** | Intercept | 3.12 | 0.39 | > 0.001 |
|  | Degree centrality | 0.30 | 0.35 | 0.386 |
|  | Timepoint (T3) | 0.99 | 0.62 | 0.117 |
|  | Degree centrality x Timepoint (T3) | -0.40 | 0.53 | 0.446 |

| *Table S3*  *Outputs of linear mixed-effects models predicting social connection outcomes (number of friends, befriended by others, reciprocal friends, reciprocal best friends, degree centrality) as a function of dance performance across timepoints (T1 – T3), including coefficients for its main effect at T1 and interactions with timepoint.* | | | | |
| --- | --- | --- | --- | --- |
| **Outcome** | **Predictor** | **Estimate** | **Std. Error** | **p-value** |
| **Friends** | Intercept | 9.94 | 1.43 | < 0.001*** |
|  | Dance performance (T1) | -65.52 | 31.99 | 0.047* |
|  | Timepoint (T2) | 0.04 | 1.23 | 0.975 |
|  | Timepoint (T3) | 1.05 | 1.31 | 0.428 |
|  | Dance performance x Timepoint (T2) | 90.03 | 53.52 | 0.101 |
|  | Dance performance x Timepoint (T3) | 56.98 | 66.12 | 0.394 |
| **Befriended by others** | Intercept | 9.43 | 0.68 | **<0.001***** |
|  | Dance performance (T1) | -5.07 | 15.17 | 0.74 |
|  | Timepoint (T2) | 0.81 | 0.58 | 0.170 |
|  | Timepoint (T3) | 1.44 | 0.62 | **0.024**** |
|  | Dance performance x Timepoint (T2) | -0.91 | 25.37 | 0.972 |
|  | Dance performance x Timepoint (T3) | 6.24 | 31.34 | 0.843 |
| **Reciprocal friends** | Intercept | 4.61 | 0.72 | **<0.001***** |
|  | Dance performance (T1) | -27.73 | 14.81 | 0.069 |
|  | Timepoint (T2) | 0.24 | 0.56 | 0.666 |
|  | Timepoint (T3) | 1.66 | 0.60 | **0.008**** |
|  | Dance performance x Timepoint (T2) | 41.06 | 24.66 | 0.102 |
|  | Dance performance x Timepoint (T3) | 48.97 | 30.49 | 0.117 |
| **Reciprocal best friends** | Intercept | 0.39 | 0.16 | **0.021*** |
|  | Dance performance (T1) | 15.27 | 7.01 | 0.035* |
|  | Timepoint (T2) | 0.70 | 0.18 | **<0.001***** |
|  | Timepoint (T3) | 1.17 | 0.18 | **<0.001***** |
|  | Dance performance x Timepoint (T2) | -14.94 | 9.17 | 0.112 |
|  | Dance performance x Timepoint (T3) | -8.34 | 10.68 | 0.44 |
| **Degree centrality** | Intercept | 1.04 | 0.09 | **<0.001***** |
|  | Dance performance (T1) | -2.79 | 2.24 | 0.221 |
|  | Timepoint (T2) | -0.01 | 0.09 | 0.884 |
|  | Timepoint (T3) | 0.12 | 0.09 | 0.196 |
|  | Dance performance x Timepoint (T2) | 2.95 | 3.78 | 0.44 |
|  | Dance performance x Timepoint (T3) | 2.69 | 4.67 | 0.568 |

| *Table S4*  *Descriptive statistics of the social network at each timepoint (T1 – T3)* | | | |
| --- | --- | --- | --- |
| **Metric** | **T1** | **T2** | **T3** |
| **Density** | 0.383 | 0.410 | 0.432 |
| **Average Path Length** | 1.71 | 1.64 | 1.58 |
| **Modularity** | 0.129 | 0.114 | 0.106 |
